# Dr. Thomas J. King

**Published:** 1889-12

**Authors:** 


### Obituary.

Dr. Thomas J. King, a prominent physician of the "Southern Tier," died at his home in Machias, Cattaraugus county, N. Y., Tuesday night, November 5, 1889, aged 64 years. Dr. King occupied an enviable position in the community in which he lived and practised, was respected by his friends and neighbors as a man of worth, enjoyed the confidence of a large clientele, and stood high among his professional compeers as an able physician. His funeral was attended by a large concourse of people, who thus testified their affection for his memory, and appropriate memorials were adopted by the several professional and civic bodies of which he was a valued member.

